# As through a Smoked Glass—Darkly

**Published:** 1886-06

**Authors:** 


### "AS THROUGH A SMOKED GLASS, DARKLY."

The *New York Medical Record* speaks of the Philadelphia Medical and Surgical Reporter's "sunset cover:"

The defeat of Dr. Shrady's revolutionary schemes and the setting down on his Philadelphia friends by the A. M. A. must have jaundiced his vision very much indeed, to cause him to see *pumpkin colored* sunsets!

